# Effects of Uncertainty on ERPs to Emotional Pictures Depend on Emotional Valence

**DOI:** 10.3389/fpsyg.2015.01927

**Published:** 2015-12-22

**Authors:** Huiyan Lin, Hua Jin, Jiafeng Liang, Ruru Yin, Ting Liu, Yiwen Wang

**Affiliations:** ^1^Key Research Base of Humanities and Social Sciences of the Ministry of Education, Center of Cooperative Innovation for Assessment and Promotion of National Mental Health, Academy of Psychology and Behavior, Tianjin Normal UniversityTianjin, China; ^2^Institute of Medical Psychology and Systems Neuroscience, University of MuensterMuenster, Germany; ^3^Center for Studies of Psychological Application, School of Psychology, South China Normal UniversityGuangzhou, China; ^4^School of Education, Guangdong University of EducationGuangzhou, China

**Keywords:** uncertainty, emotion, valence, P2, N2, LPP, ERPs

## Abstract

Uncertainty about the emotional content of an upcoming event has found to modulate neural activity to the event before its occurrence. However, it is still under debate whether the uncertainty effects occur after the occurrence of the event. To address this issue, participants were asked to view emotional pictures that were shortly after a cue, which either indicated a certain emotion of the picture or not. Both certain and uncertain cues were used by neutral symbols. The anticipatory phase (i.e., inter-trial interval, ITI) between the cue and the picture was short to enhance the effects of uncertainty. In addition, we used positive and negative pictures that differed only in valence but not in arousal to investigate whether the uncertainty effect was dependent on emotional valence. Electroencephalography (EEG) was recorded during the presentation of the pictures. Event-related potential (ERP) results showed that negative pictures evoked smaller P2 and late LPP but larger N2 in the uncertain as compared to the certain condition; whereas we did not find the uncertainty effect in early LPP. For positive pictures, the early LPP was larger in the uncertain as compared to the certain condition; however, there were no uncertainty effects in some other ERP components (e.g., P2, N2, and late LPP). The findings suggest that uncertainty modulates neural activity to emotional pictures and this modulation is altered by the valence of the pictures, indicating that individuals alter the allocation of attentional resources toward uncertain emotional pictures dependently on the valence of the pictures.

## Introduction

From a biological point of view, anticipating the emotional content of an upcoming event according to environmental cues may help individuals in preparing adaptive reactions to approach the benefit and to avoid the harm. However, people are living in an ever-changing world; there is often uncertainty about which emotional content of the event will actually occur. This uncertainty has been found to be associated with anxiety (Grupe and Nitschke, 2013). Identifying the processing of uncertainty may contribute to understanding the detrimental effects of uncertainty on well-being and psychological symptoms.

Studies have indicated that uncertainty modulates neural activity to emotional pictures before their occurrences (during the anticipation period; e.g., Onoda et al., 2007; Lin et al., 2014a). In our previous Event-related potential (ERP) study (Lin et al., 2014a), for example, neutral symbols (e.g., arrows; cues) that uncertainly as compared to certainly signify the emotional content of the upcoming picture generally evoked larger N2 amplitudes. When the following picture was negative, early contingent negative variation (CNV)^1^ was reduced for uncertain as compared to certain cues.

Meanwhile, several studies investigated the uncertainty effects after the occurrences of the emotional pictures. However, results are under debate in both functional magnetic resonance imaging (fMRI) and electroencephalography (EEG) studies. In terms of the fMRI studies, Sarinopoulos et al. (2010) showed that neural activity in insula and amygdala was stronger for negative pictures following uncertain as compared to certain cues; whereas this uncertainty effect was not found in Onoda et al.'s (2008) study, regardless of the emotional contents (positive and negative) of the pictures. For EEG studies, Onoda et al.'s (2006) visual evoked magnetic fields (VEF) study found larger M120 amplitudes for uncertain compared to certain negative pictures. Using the same paradigm, however, uncertain and certain emotional (positive and negative) pictures were found to have similar lower-1, lower-2, and upper alpha^2^ in event-related desynchronization/synchronization (ERD/ERS; Onoda et al., 2007). Accordingly, the authors suggested that the sensory and cognitive processing of emotional pictures are unaffected by uncertainty (Onoda et al., 2007).

In the above-mentioned fMRI studies (Onoda et al., 2008; Sarinopoulos et al., 2010), the temporal resolution of BOLD fMRI was ~ 2 s (i.e., TR = 2 s). However, the effect of uncertainty on emotional stimuli seems to occur only in the first second relative to the onset of the stimuli (Onoda et al., 2006; Gole et al., 2012; Yang et al., 2012). In this case, the temporal resolution may be not high enough to precisely and accurately capture the time course of the uncertainty effect, which results in altering the uncertainty effects. Regarding to EEG studies, while VEF and ERD/ERS are thought to have high temporal resolution, it is still unclear whether the indices (i.e., lower-1, lower-2, and upper alpha) used in Onoda et al.'s (2006, 2007) studies are suitable to investigate the effects related to emotional anticipation. Emotional pictures following anticipatory cues as compared to these pictures without any anticipatory cues were shown to be similar in VEF (Onoda et al., 2006) and to be differential in lower-2 alpha only at late stages of processing (e.g., >300 ms; Onoda et al., 2007). Using ERPs that also have high temporal resolution, however, we found the anticipation effects at both early and late stages (Lin et al., 2012, 2014b).

Due to temporal resolution and indices, ERPs are ideal to investigate neural responses during the perception of pictures which emotional contents are certain or uncertain. To the best of our knowledge, two ERP studies have investigated whether uncertainty modulates the processing of emotional pictures (Gole et al., 2012; Yang et al., 2012). Gole et al. (2012) showed that emotionally uncertain compared to certain pictures evoked greater early posterior negativity (EPN; overlapping with N2) and smaller late positive potential (LPP) amplitudes, regardless of the emotional contents (negative and neutral) of the pictures. Using facial pictures only, Yang et al. (2012) found larger P2 amplitudes for emotionally (fearful and neutral) uncertain as compared to certain faces. For fearful faces, the N2 amplitudes were smaller after uncertain as compared to certain cues.

In Gole et al.'s (2012) study, however, there was a long anticipatory phase (i.e., inter-trial interval, ITI) between the cue and the picture (6 s). Participants may have enough time to prepare for the upcoming event during the phase, which may reduce the effect of uncertainty (Lin et al., 2014a). Different from previous studies in which certain and uncertain cues were used by neutral symbols (Onoda et al., 2006, 2007, 2008; Sarinopoulos et al., 2010; Gole et al., 2012); Yang et al. (2012) used emotional pictures and neutral symbols (i.e., “+”) as certain and uncertain cues, respectively. For fearful faces, certain and uncertain cues differed not only in the meanings but also in some other factors (e.g., the emotional contents and composition of the cues). Therefore, it is still unclear whether the uncertainty effects observed in the study were relevant in the meanings of the cues or these factors.

Therefore, our present study aimed to further investigate whether uncertainty about the emotional content of an upcoming picture modulates the ERPs to the picture. To address this issue, EEG was recorded while participants viewed emotional pictures. Each trial started with a cue, followed by an anticipatory interval and subsequently, a picture. The cue either indicated the emotional content of the following picture or not. All the cues were used by neutral symbols (e.g., arrows). The ITI between the cue and the picture was short (about 2 s) to enhance the effect of uncertainty. Furthermore, as emotional valence and arousal may influence the uncertainty effect and we were interested in the modulation of valence on the uncertainty effect, the present study used positive and negative pictures which differed in valence but not in arousal. Based on the above-mentioned studies, we expected that uncertain as compared to certain emotional pictures might evoke greater P2 and smaller LPP amplitudes. However, it was still unclear whether uncertainty modulates the N2 amplitudes for emotional pictures.

## Methods

### Participants

Nineteen undergraduate and postgraduate students (11 females and 8 males; age: *M* ± *SD* = 22.42 ± 1.85) were recruited in South China Normal University via advertisements in return for the compensation of 30 RMB for this study. All participants were right-handed, as assessed through the Edinburgh Handedness Inventory (Oldfield, 1971). Participants had normal or corrected-to-normal vision. No participants reported any medical, neurological or psychiatric disorders. Participants were told that the study was to investigate the neural activity to emotional pictures and written informed consent was given prior to the experiment. Ethical approval was obtained from the Ethics Committee of School of Psychology, South China Normal University.

### Stimuli

Stimuli were the same as those in our previous studies (Lin et al., 2012, 2014a,b). Stimuli were 160 colored pictures (80 positive and 80 negative) that were obtained from Chinese Affective Picture System (CAPS; Bai et al., 2005). All the pictures were converted to gray-level, were of the same size (11 × 8 cm, 6.30 × 4.58°) and resolution (100 pixels per inch) and were aligned in the non-emotional features (e.g., luminance, contrast, and composition).

The pictures were rated on valence and arousal using a 9-point scale ranging from “1” (extremely unpleasant) to “9” (extremely pleasant) and “1” (low arousal) to “9” (high arousal), respectively, by another 22 undergraduate and postgraduate students (11 females, 19–25 years, *M* ± *SD* = 21.24 ± 1.75). The ratings of positive and negative pictures showed significant difference in valence [*t*_(21)_ = 10.55, *p* = 7.48E-10, 6.88 ± 1.03 vs. 3.06 ± 1.28], but not in arousal [*t*_(21)_ = −0.03, *ps.* > 0.05, 5.36 ± 1.51 vs. 5.38 ± 1.77].

### Procedure

Participants were seated in a soundproof and dimly room approximately 1 m directly in front of a computer monitor. Stimuli were presented using E-prime 1.0 software (Psychology Software Tools, Inc., Pittsburgh, PA, USA) on a black screen in the center of a 17″ monitor with a screen resolution of 1024 by 768 pixels. All stimuli were presented against a gray background. Every trial started with a black fixation cross for 500 ms, replaced by a random blank screen varying from 1000 to 2000 ms (*M* = 1500 ms). A bidirectional, a left or a right arrow (1.03 × 0.23°) was randomly presented for 200 ms. The bidirectional arrow served as the uncertain cue in that it was randomly followed by a positive or negative picture with equal conditional probabilities. The right and the left arrow served as the certain cue for a subsequent positive and a negative picture, respectively. A positive or a negative picture was shown at the center of the screen for 1000 ms after another blank screen of random duration between 1600 and 2000 ms (*M* = 1800 ms). Participants were told the meaning of the cue before the formal experiment and were asked to view the cues and the pictures during their presentations. After another blank screen was shown for 200 ms following the picture, participants were asked to rate the pleasantness of the picture on a 9-point scale ranging from “1” (extremely unpleasant) to “9” (extremely pleasant). There was no time limit for the response. The subsequent trial started after another blank screen for 1500 ms.

Each picture described in the Stimuli Section was used twice, once after uncertain cues and once after certain cues. That is, the task comprised a total of 320 trials. Before the formal experiment, there were 20 trials of practice to familiarize with the experimental procedure. The pictures used in the formal experiment were not used in the practice.

### EEG recording

EEG was continuously recorded using a NEUROSCAN Synamps2 AC-amplifier (Neuroscan, Inc., Sterling, VA, USA). The Ag/AgCl electrodes were placed upon the scalp by a 32 channel Quick-Cap in accordance with the international extended 10–20 system (FP1, FP2, F7, F3, Fz, F4, F8, FT7, FC3, FCz, FC4, FT8, T7, C3, Cz, C4, T8, TP7, CP3, CPz, CP4, TP6, P7, P3, Pz, P4, P8, O1, Oz, and O2). EEG electrodes were connected to ground and were referenced to the right mastoid online. The horizontal electrooculogram (EOG) was recorded from two electrodes at the outer canthi of both eyes, and the vertical EOG was monitored bipolarly from electrodes above and below the left eye. The EEG was amplified using a 0.05–100 Hz band-pass and sampled at 1000 Hz/channel with a 50 Hz notch filter. Electrode impedances were maintained below 5 kΩ.

The EEG data were analyzed offline using the SCAN 4.3 software. Raw EEG signals were digitally re-referenced to the average of two mastoids. Ocular movements were inspected and removed based on the default parameter of the SCAN 4.3 ocular artifact tool. EEG was then segmented from −200 ms until 1000 ms relative to the onset of the picture, with the first 200 ms serving as a baseline. Artifact rejection was carried out using an amplitude threshold of 100 μV. Trials were averaged separately for each channel and each experimental condition and averaged ERPs were digitally low-pass filtered at 30 Hz (24 db/oct, zero phase shift, Butterworth).

ERPs were quantified using mean amplitudes for P2 (130–180 ms), N2 (220–300 ms), early LPP (350–450 ms), and late LPP (550–1000 ms). P2 and N2 were measured at frontal (F3, Fz, F4), frontal-central (FC3, FCz, FC4), and central (C3, Cz, C4) electrodes. Early and late LPP were measured at frontal (F3, Fz, F4), frontal-central (FC3, FCz, FC4), central (C3, Cz, C4), central-parietal (CP3, CPz, CP4), and parietal (P3, Pz, P4) electrodes. The time window for P2 was chosen to correspond with peaks identified in the grand waveforms across all conditions (155 ms) and previous studies (Gole et al., 2012; Lin et al., 2012, 2014b) and time windows for N2, early, and late LPP were chosen based on visual inspection of the grand waveforms and previous studies (Gole et al., 2012; Lin et al., 2012, 2014b). The electrodes of interest for all the components were based on visual inspection of the grand waveforms and previous studies (Gole et al., 2012; Lin et al., 2012, 2014b; Giglio et al., 2013; Richards et al., 2013). The average number of trials was shown in Table 1.

**Table 1:** **Mean number of trials for each experimental condition**.

**Uncertain positive**	**Certain positive**	**Uncertain negative**	**Certain negative**
72.42	71.63	74.11	74.37

### Data analysis

**Figure 1:**
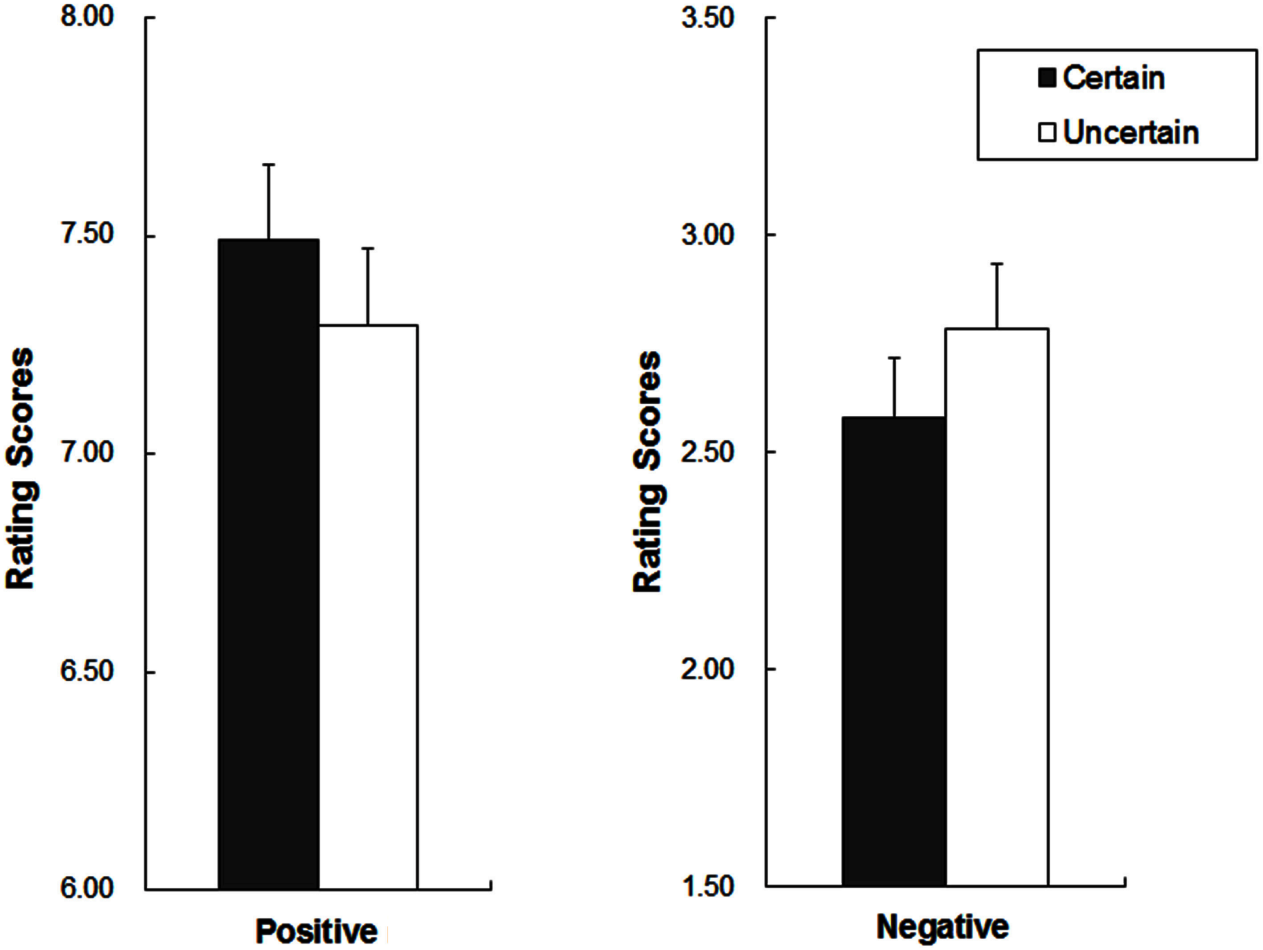
**Behavioral results**. Mean (rectangles) and standard deviation (vertical lines) for the ratings in the certain and the uncertain condition (left for positive pictures and right for negative pictures).

For statistical analysis of behavioral results, the ratings were analyzed in 2 × 2 repeated measures analyses of variance (ANOVAs) with uncertainty (uncertain vs. certain) and emotion (positive vs. negative) as within-subject factors. Mean ratings and their *SD* across conditions are presented in Figure 1.

**Figure 2:**
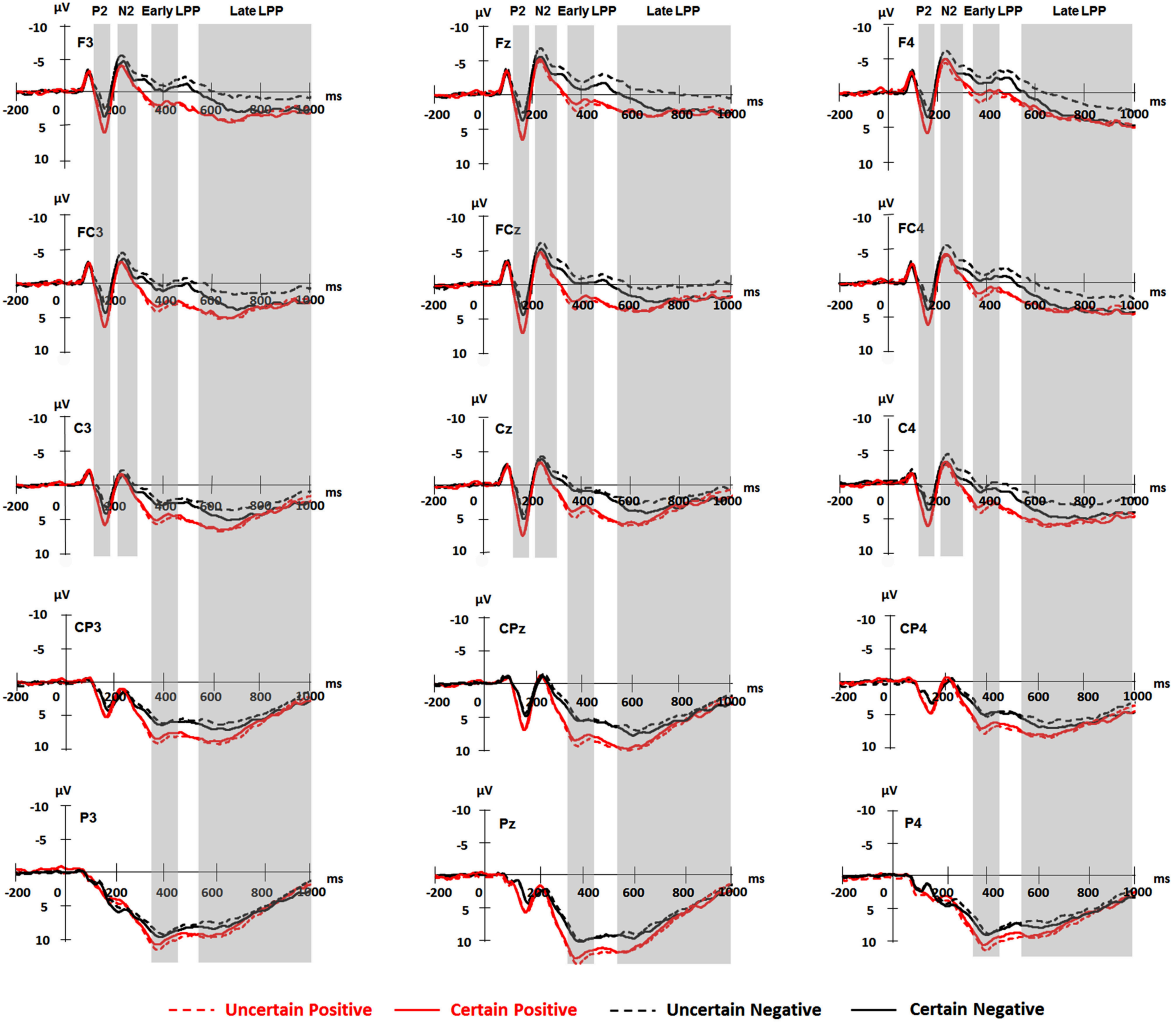
**Grand averaged ERPs for frontal (F3, Fz, F4), frontal-central (FC3, FCz, FC4), central (C3, Cz, C4), central-parietal (CP3, CPz, CP4), and parietal (P3, Pz, P4) scalp sites elicited by pictures in each experimental condition**. Shaded areas correspond to the time window for P2 (130–180 ms), N2 (220–300 ms), early (350–450 ms), and late LPP (550–1000 ms).

**Figure 3:**
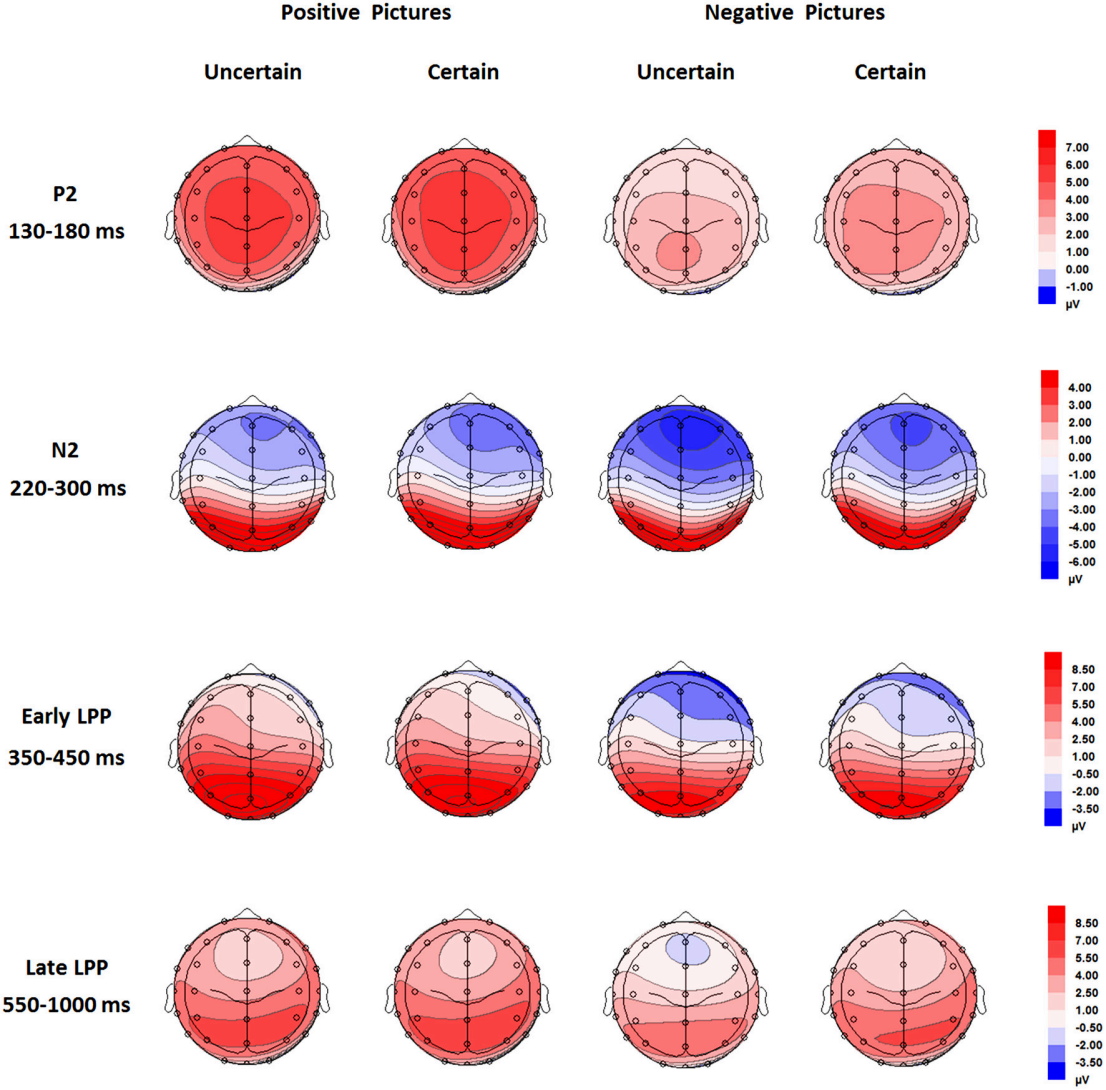
**Topographical maps based on mean amplitudes of P2 (130-180 ms), N2 (220–300 ms), early (350–450 ms), and late LPP (550–1000 ms) for all the experimental conditions**.

For ERPs, the above-mentioned ANOVAs were performed separately for P2, N2, early, and late LPP. The analysis for P2 and N2 included site (Frontal vs. Frontal-Central vs. Central) and hemisphere (Left vs. Middle vs. Right) as within-subject factors. The analysis for early and late LPP included site (Frontal vs. Frontal-Central vs. Central vs. Central-Parietal vs. Parietal) and hemisphere (Left vs. Middle vs. Right) as within-subject factors. Grand-average waveforms and topographical maps of all the components are presented in Figures 2, 3, respectively. *M* and *SD* of the mean amplitudes for these components are presented in Tables 2–5.

**Table 2:** **Mean (*M*) and standard deviation (*SD*) for P2 mean amplitudes (μV)**.

	**Uncertain positive**		**Certain positive**		**Uncertain negative**		**Certain negative**	
	***M***	***SD***	***M***	***SD***	***M***	***SD***	***M***	***SD***
F3	4.65	4.94	4.72	4.91	1.55	4.44	2.67	5.18
Fz	4.80	5.64	4.88	5.60	1.58	4.99	2.62	5.73
F4	4.66	5.59	4.65	5.37	1.60	4.96	2.65	5.64
FC3	4.83	5.08	4.85	5.07	1.91	4.49	2.99	5.26
FCz	5.31	5.84	5.37	5.68	2.08	5.13	3.11	5.89
FC4	4.62	5.75	4.75	5.43	1.78	5.09	2.85	5.67
C3	4.59	4.42	4.56	4.58	2.17	3.95	3.06	4.83
Cz	5.89	5.80	5.84	5.80	2.97	5.06	3.60	5.89
C4	4.49	5.40	4.49	4.94	0.97	7.19	2.68	5.26

**Table 3:** ***M* and *SD* for N2 mean amplitudes (μV)**.

	**Uncertain positive**		**Certain positive**		**Uncertain negative**		**Certain negative**	
	***M***	***SD***	***M***	***SD***	***M***	***SD***	***M***	***SD***
F3	−4.19	3.37	−4.45	3.69	−5.85	3.41	−4.97	3.85
Fz	−5.23	4.17	−5.73	4.27	−7.16	4.01	−6.01	4.37
F4	−4.65	4.44	−5.43	4.56	−6.60	4.53	−5.41	4.63
FC3	−3.42	3.62	−3.68	3.96	−4.74	3.50	−3.96	4.25
FCz	−4.96	4.52	−5.33	4.63	−6.60	4.19	−5.72	4.88
FC4	−4.28	4.63	−4.76	4.67	−5.90	4.66	−4.66	4.73
C3	−1.80	3.89	−2.02	4.37	−2.48	3.65	−1.88	4.83
Cz	−3.47	5.16	−3.93	5.21	−4.46	4.56	−3.77	5.38
C4	−2.90	4.97	−3.72	5.00	−5.71	7.42	−3.12	5.18

**Table 4:** ***M* and *SD* for early LPP mean amplitudes (μV)**.

	**Uncertain positive**		**Certain positive**		**Uncertain negative**		**Certain negative**	
	***M***	***SD***	***M***	***SD***	***M***	***SD***	***M***	***SD***
F3	2.40	5.50	1.72	5.04	−1.32	5.46	−0.52	5.48
Fz	1.91	6.35	0.95	5.77	−2.26	5.90	−1.24	6.04
F4	1.12	5.97	0.01	5.40	−2.33	5.86	−1.54	5.85
FC3	3.67	5.52	2.90	5.04	−0.06	5.33	0.67	5.51
FCz	2.95	6.18	2.01	5.48	−1.43	5.57	−0.55	5.95
FC4	1.91	5.72	1.11	4.98	−1.52	5.40	−0.63	5.44
C3	5.23	4.61	4.55	4.28	1.94	4.57	2.58	5.16
Cz	4.77	6.25	3.84	5.60	0.51	5.31	1.03	5.88
C4	3.45	4.75	2.78	4.27	−0.97	6.55	1.29	5.18
CP3	8.34	4.84	7.87	4.84	5.15	4.60	5.89	5.48
CPz	8.48	5.88	7.71	5.72	4.63	5.29	5.07	6.47
CP4	6.96	4.76	6.32	4.75	3.95	4.57	4.50	5.46
P3	10.80	5.22	10.59	5.23	8.55	5.14	9.09	5.56
Pz	12.14	6.40	11.72	6.19	9.04	5.65	9.44	6.48
P4	10.03	4.64	9.68	4.74	7.96	4.45	8.39	5.17

**Table 5:** ***M* and *SD* for late LPP mean amplitudes (μV)**.

	**Uncertain positive**		**Certain positive**		**Uncertain negative**		**Certain negative**	
	***M***	***SD***	***M***	***SD***	***M***	***SD***	***M***	***SD***
F3	3.64	3.78	3.98	4.24	1.09	3.38	2.75	3.91
Fz	2.76	3.82	2.85	4.15	−0.03	3.48	2.06	4.10
F4	3.86	3.50	3.58	4.24	1.36	3.64	3.26	4.67
FC3	4.00	3.69	4.10	4.57	1.52	3.24	3.14	3.79
FCz	2.63	3.98	2.78	4.57	0.38	3.08	2.05	4.04
FC4	3.76	3.56	3.95	3.88	1.57	3.37	3.50	4.29
C3	4.70	2.98	4.81	3.63	2.93	2.98	4.03	3.48
Cz	4.03	4.24	4.20	4.75	2.08	3.98	3.14	4.65
C4	5.21	3.92	5.34	4.19	2.31	4.06	5.04	4.94
CP3	6.19	2.77	6.31	3.39	4.38	2.75	5.34	3.36
CPz	6.18	4.34	6.22	4.60	4.68	3.89	5.32	5.03
CP4	6.29	3.47	6.40	3.73	4.68	3.40	5.82	4.47
P3	6.14	3.02	6.44	3.56	5.14	2.93	5.70	3.60
Pz	6.18	4.65	6.61	4.91	5.44	4.23	5.80	4.81
P4	5.58	3.53	5.84	4.21	4.82	3.70	5.45	4.49

Degree of freedom and *p*-values of repeated measurements were corrected by Greenhouse-Geisser and *p*-values of *post-hoc* tests were corrected by Bonferroni correction. Please note that the effects which failed to reach statistical significance would not be reported (*ps.* > 0.05).

## Results

### Behavioral results

The analysis showed a significant main effect of emotion [*F*_(1, 18)_ = 289.85, *p* = 1.52E-12, $\eta_{p}^{2}=0.94$], with larger ratings for positive than negative pictures. The interaction between emotion and uncertainty was also significant [*F*_(1, 18)_ = 17.37, *p* = 0.001, $\eta_{p}^{2}=0.49$]. For positive pictures, the ratings were higher in the certain as compared to the uncertain condition [*t*_(18)_ = 4.34, *p* = 4.00E-4]; for negative pictures, however, the ratings were higher in the uncertain as compared to the certain condition [*t*_(18)_ = −3.47, *p* = 0.003].

### ERP results

#### P2 components

The analysis of P2 amplitudes revealed main effects of uncertainty [*F*_(1, 18)_ = 4.97, *p* = 0.039, $\eta_{p}^{2}=0.22$], emotion [*F*_(1, 18)_ = 67.09, *p =* 1.75E-7, $\eta_{p}^{2}=0.79$], and hemisphere [*F*_(2, 36)_ = 4.15, *p* = 0.024, $\eta_{p}^{2}=0.19$]. The P2 amplitudes were larger for pictures in the certain as compared to the uncertain condition and for positive compared with negative pictures. The amplitudes were larger for the Middle as compared to the Right (*p* = 0.010), but they were similar in the Left as compared to the Middle and in the Left compared to the Right (*ps.* > 0.05).

There was a significant interaction between site and hemisphere [*F*_(2, 30)_ = 6.01, *p* = 0.010, $\eta_{p}^{2}=0.25$]. No hemisphere effect was found at the Frontal and the Frontal-Central site (*ps.* > 0.05). For the Central site, the effect of hemisphere was significant [*F*_(2, 36)_ = 6.68, *p* = 0.003, $\eta_{p}^{2}=0.27$], with larger amplitudes for the Middle compared to the Left (*p* = 0.025), and for the Middle compared to the Right (*p* = 0.008).

The interaction between uncertainty and emotion was significant [*F*_(1, 18)_ = 10.32, *p* = 0.005, $\eta_{p}^{2}=0.36$]. Further analyses showed that for negative pictures, the amplitudes were larger in the certain compared to the uncertain condition [*t*_(18)_ = 3.32, *p* = 0.004]; for positive pictures, however, the effect of uncertainty did not reach statistical significance (*ps.* > 0.05).

#### N2 components

The ANOVA for N2 amplitudes showed main effects of emotion [*F*_(1, 18)_ = 16.99, *p* = 0.001, $\eta_{p}^{2}=0.49$], site [*F*_(1, 20)_ = 18.77, *p* = 2.00E-4, $\eta_{p}^{2}=0.51$], and hemisphere [*F*_(2, 36)_ = 9.51, *p* = 5.00E-4, $\eta_{p}^{2}=0.35$]. Negative pictures elicited larger amplitudes than did positive pictures. Pictures were larger in amplitudes at the Frontal as compared to the Frontal-Central (*p* = 0.048) and the Central (*p* = 0.001) site and at the Frontal-Central as compared to the Central site (*p* = 2.00E-4). The amplitudes were also larger at the Middle as compared to the Left (*p* = 5.00E-4).

Furthermore, there was an interaction between uncertainty and emotion [*F*_(1, 18)_ = 9.32, *p* = 0.007, $\eta_{p}^{2}=0.34$]. Follow-up analyses showed larger amplitudes for negative pictures preceded by uncertain as compared to certain cues [*t*_(18)_ = 2.66, *p* = 0.016], but this uncertainty effect did not reach statistical significance for positive pictures (*ps.* > 0.05).

#### Early LPP components

The analysis showed a main effect of emotion [*F*_(1, 18)_ = 59.95, *p* = 3.89E-7, $\eta_{p}^{2}=0.77$], with larger amplitudes for positive as compared to negative pictures. The main effect of site was significant [*F*_(1, 22)_ = 71.57, *p* = 5.24E-9, $\eta_{p}^{2}=0.80$]. The amplitudes were larger for the parietal site as compared to the other sites (Frontal: *p* = 2.86E-7; Frontal-Central: *p* = 2.95E-7; Central: *p* = 4.25E-8; Central-Parietal: *p* = 1.04E-7), for the Central-Parietal site as compared to the Frontal (*p* = 1.10E-5), Frontal-Central (*p* = 1.00E-5), and Central sites (*p* = 1.00E-6), for the Central site as compared to the Frontal (*p* = 0.001) and Frontal-Central sites (*p* = 0.004) and for the Frontal-Central as compared to the Frontal site (*p* = 0.001). While the main effect of hemisphere was also significant [*F*_(2, 36)_ = 3.63, *p* = 0.037, $\eta_{p}^{2}=0.17$], pairwise comparisons did not show any significant effects among all conditions (*ps.* > 0.05).

There was an interaction between uncertainty and emotion [*F*_(1, 18)_ = 5.58, *p* = 0.030, $\eta_{p}^{2}=0.24$]. For positive pictures, the early LPP was larger in the uncertain as compared to the certain condition [*t*_(18)_ = −2.37, *p* = 0.029, $\eta_{p}^{2}=0.24$]. For negative pictures, however, the effect of uncertainty did not reach statistical significance (*ps.* > 0.05).

The interaction between emotion and site was significant [*F*_(1, 24)_ = 5.33, *p* = 0.023, $\eta_{p}^{2}=0.23$]. The early LPP was more shifted in the positive direction for positive as compared to negative pictures at all sites, though to different extents [Frontal: *t*_(18)_ = 7.96, *p* = 2.61E-7; Frontal-Central: *t*_(18)_ = 8.36, *p* = 1.31E-7; Central: *t*_(18)_ = 7.70, *p* = 4.22E-7; Central-Parietal: *t*_(18)_ = 7.04, *p* = 1.00E-6; Parietal: *t*_(18)_ = 4.92, *p* = 1.10E-4].

The interaction between emotion and hemisphere was significant [*F*_(1, 24)_ = 6.91, *p* = 0.009, $\eta_{p}^{2}=0.28$]. The early LPP amplitudes were larger for positive as compared to negative pictures at all levels of hemisphere, though to different extents [Left: *t*_(18)_ = 7.34, *p* = 8.23E-7; Middle: *t*_(18)_ = 7.87, *p* = 3.10E-7; Right: *t*_(18)_ = 6.60, *p* = 3.00E-6].

We also found an interaction between site and hemisphere [*F*_(4, 78)_ = 3.00, *p* = 0.021, $\eta_{p}^{2}=0.14$]. Separate analysis for each hemisphere showed that the site effect was significant at all levels of hemisphere, though to different extents [Left: *F*_(1, 25)_ = 54.05, *p* = 1.45E-8, $\eta_{p}^{2}=0.75$; Middle: *F*_(1, 23)_ = 59.20, *p* = 1.44E-8, $\eta_{p}^{2}=0.77$; Right: *F*_(1, 27)_ = 77.36, *p* = 7.78E-11, $\eta_{p}^{2}=0.81$]. For the Left, the early LPP was larger for the Parietal site as compared to the other sites [Frontal: *p* = 1.00E-6; Frontal-Central: *p* = 2.00E-6; Central: *p* = 8.71E-7; Central-Parietal: *p* = 8.00E-5], for the Central-Parietal site as compared to the Frontal (*p* = 3.70E-5), Frontal-Central (*p* = 7.90E-5), and Central sites (*p* = 1.20E-5), for the Central site as compared to the Frontal (*p* = 4.49E-4) and Frontal-Central sites (*p* = 0.003) and for the Frontal-Central as compared to the Frontal site (*p* = 0.002). For the Middle, the early LPP was larger for the Parietal site as compared to the other sites (Frontal: *p* = 1.00E-6; Frontal-Central: *p* = 6.33E-7; Central: *p* = 1.15E-7; Central-Parietal: *p* = 2.66E-7), for the Central-Parietal site as compared to the Frontal (*p* = 6.40E-5), Frontal-Central (*p* = 3.60E-5), and Central sites (*p* = 4.00E-5), for the Central site as compared to the Frontal (*p* = 0.007) and Frontal-Central sites (*p* = 0.009) and for the Frontal-Central as compared to the Frontal site (*p* = 0.043). For the Right, the early LPP was larger for the Parietal site as compared to the Frontal (*p* = 1.15E-7), Frontal-Central (*p* = 1.15E-7), Central (*p* = 4.64E-8), and Central-Parietal sites (*p* = 4.07E-7), for the Central-Parietal site as compared to the Frontal (*p* = 3.00E-6), Frontal-Central (*p* = 2.00E-6), and Central sites (*p* = 9.64E-7) and for the Central (*p* = 0.008) and Frontal-Central (*p* = 0.012) sites as compared to the Frontal site.

#### Late LPP components

The AVONA showed main effects of anticipation [*F*_(1, 18)_ = 5.43, *p* = 0.032, $\eta_{p}^{2}=0.23$], emotion [*F*_(1, 18)_ = 11.54, *p* = 0.003, $\eta_{p}^{2}=0.39$], and site [*F*_(1, 22)_ = 16.19, *p* = 2.74E-4, $\eta_{p}^{2}=0.47$]. The late LPP was more shifted in the positive direction in the certain as compared to the uncertain condition and for positive as compared to negative pictures. The late LPP was also larger for the Parietal site as compared to the Frontal (*p* = 0.017), Frontal-Central (*p* = 0.008), and Central sites (*p* = 0.050), for the Central-Parietal site as compared to the Frontal (*p* = 0.002), Frontal-Central (*p* < 0.001), and Central sites (*p* = 0.002) and for the Central site as compared to the Frontal (*p* = 0.042) and Frontal-Central sites (*p* = 0.003).

The interaction between emotion and site was significant [*t*_(1, 24)_ = 3.97, *p* = 0.048, $\eta_{p}^{2}=0.18$]. Positive as compared to negative pictured evoked larger late LPP amplitudes at the Frontal [*t*_(18)_ = 4.13, *p* = 0.001], Frontal-Central [*t*_(18)_ = 3.43, *p* = 0.003], Central [*t*_(18)_ = 3.30, *p* = 0.004], Central-Parietal sites [*t*_(18)_ = 3.00, *p* = 0.008], thought to different extents. However, the emotional effect was not significant at the Parietal site (*ps.* > 0.05).

The interaction between site and hemisphere showed statistical significance [*F*_(4, 72)_ = 4.54, *p* = 0.002, $\eta_{p}^{2}=0.20$]. Separate analysis for each hemisphere showed that the site effect was significant at all levels of hemisphere, though to different extents [Left: *F*_(1, 23)_ = 12.65, *p* = 0.001, $\eta_{p}^{2}=0.41$; Middle: *F*_(1, 25)_ = 17.85, *p* = 8.30E-5, $\eta_{p}^{2}=0.50$; Right: *F*_(1, 27)_ = 10.90, *p* = 0.001, $\eta_{p}^{2}=0.38$]. For the Left, the late LPP was larger for the Parietal as compared to the Central (*p* = 0.035), Frontal-Central (*p* = 0.029) and Frontal (*p* = 0.024) sites and for the Central-Parietal as compared to the Central (*p* = 0.002), Frontal-Central (*p* = 0.007) and Frontal (*p* = 0.009) sites. For the Middle, the late LPP was larger for the Parietal as compared to the Central (*p* = 0.009), Frontal-Central (*p* = 0.001), and Frontal (*p* = 0.009) sites, for the Central-Parietal as compared to the Central (*p* = 0.004), Frontal-Central (*p* = 2.46E-4), and Frontal (*p* = 0.004) sites and for the Central as compared to the Frontal-Central site (*p* = 0.017). For the Right, the amplitudes were larger for the Central-Parietal as compared to the Central (*p* = 0.029), Frontal-Central (*p* = 0.001), and Frontal sites (*p* = 0.001) and for the Central site as compared to the Frontal-Central (*p* = 0.013) and Frontal (*p* = 0.036) sites.

More importantly, the three-way interaction among uncertainty, emotion, and site was also significant [*F*_(2, 40)_ = 9.48, *p* = 2.89E-4, $\eta_{p}^{2}=0.35$]. Separate analysis for each site showed that for the Frontal and the Frontal-Central site, the late LPP was larger in the certain as compared to the uncertain condition [Frontal: *F*_(1, 18)_ = 6.05, *p* = 0.024, $\eta_{p}^{2}=0.25$; Frontal-Central: *F*_(1, 18)_ = 7.10, *p* = 0.016, $\eta_{p}^{2}=0.28$] and for positive as compared to negative pictures [Frontal: *F*_(1, 18)_ = 17.01, *p* = 0.001, $\eta_{p}^{2}=0.49$; Frontal-Central: *F*_(1, 18)_ = 11.77, *p* = 0.003, $\eta_{p}^{2}=0.40$]. More importantly, the interaction between uncertainty and emotion was significant [Frontal: *F*_(1, 18)_ = 7.63, *p* = 0.013, $\eta_{p}^{2}=0.30$; Frontal-Central: *F*_(1, 18)_ = 4.75, *p* = 0.043, $\eta_{p}^{2}=0.21$]. For negative pictures, the late LPP was larger in the certain as compared to the uncertain condition [Frontal: *t*_(18)_ = 3.06, *p* = 0.007; Frontal-Central: *t*_(18)_ = 2.94, *p* = 0.009]; whereas the uncertainty effect did not reach statistical significance for positive pictures (*ps.* > 0.05). For the Central site, the late LPP was generally larger in the certain as compared to the uncertain condition [*F*_(1, 18)_ = 6.26, *p* = 0.022, $\eta_{p}^{2}=0.26$] and for positive as compared to negative pictures [*F*_(1, 18)_= 10.88, *p* = 0.004, $\eta_{p}^{2}=0.38$]. In addition, there was a trend for the interaction between uncertainty and emotion [*F*_(1, 18)_ = 3.92, *p* = 0.063, $\eta_{p}^{2}=0.18$]. The late LPP was larger for certain as compared to uncertain negative pictures [*t*_(18)_ = 2.59, *p* = 0.019], whereas this effect was not significant for positive pictures (*ps.* > 0.05). For the Central-Parietal site, the analysis only showed a main effect of emotion [*F*_(1, 18)_ = 9.02, *p* = 0.008, $\eta_{p}^{2}=0.33$], with larger amplitudes for positive as compared to negative pictures. For the Parietal site, however, no main effects or interaction reach statistical significance (*ps.* >0.05).

## Discussion

The present study further investigated whether uncertainty about the emotional content of an upcoming picture modulates ERPs to the picture. Results showed that uncertain as compared to certain negative pictures evoked smaller P2 and late LPP and larger N2 amplitudes. For positive pictures, early LPP was greater in amplitude in the uncertain compared to the certain condition. Taken together, the findings suggest that uncertainty modifies the ERPs to emotional pictures and that the uncertainty effects are altered by emotional valence of the pictures.

P2 is a positive component that peaks over anterior sites around 200 ms following stimulus onset. The P2 is related to selected attention in early sensory processes (e.g., Yuan et al., 2007; van Hooff et al., 2011), with enhanced amplitudes for certain stimuli (e.g., Kanske et al., 2011). In a subsequent time range, N2 is a negative deflection over anterior scalp sites at ~ 200–300 ms. The N2 is supposed to be relevant in attention allocation in the final stages of sensory processing (Olofsson et al., 2008; Ernst et al., 2013), with larger N2 amplitudes for uncertain as compared to certain emotional pictures (Gole et al., 2012). Therefore, our findings suggest that uncertainty reduces the attention toward negative pictures during early sensory processes, but this uncertainty effect is reversed in late sensory processes.

Surprisingly, the uncertainty effects related to attention are different in different sensory processes of negative pictures. Knowing about the negative content of the upcoming picture is supposed to activate attention mechanism before the occurrence of the picture (e.g., Böcker et al., 2001; Erk et al., 2006; Lin et al., 2012, 2014a, 2015), which is facilitated in enhancing the attention toward the pictures shortly after they occurred (i.e., early sensory stages of picture processing; Lin et al., 2012). In the present study, as participants had not known about the negative content of the pictures in the preceding uncertain anticipation phase, the attentional resources allocated to uncertain negative pictures were very limited at early sensory stages of picture processing. Just at these stages, however, participants clearly knew about the emotional contents of the pictures (as the emotional effects started in the time range of P2). In order to process uncertain negative pictures in a better way, participants may enhance the allocation of attentional resources to uncertain negative pictures to a large extent in later stages of sensory processing (e.g., N2), resulting in observing the enhanced attention toward these pictures in these stages.

For positive pictures, we did not find the uncertainty effect on P2 and N2, indicating that uncertainty does not modulate attention toward positive pictures during sensory processes. Due to negativity bias, participants may overestimate the frequency of negative pictures but underestimate that of non-negative pictures in uncertain circumstances (Sarinopoulos et al., 2010; Grupe and Nitschke, 2011). The occurrences of positive pictures may slightly violate the expectations and thus, enhance the attention at early stages of sensory processing (e.g., Qin et al., 2009). Therefore, the difference in certain as compared to uncertain positive pictures is reduced. In addition, as the attention toward uncertain positive pictures has been enhanced at early stages of sensory processing, it may be unnecessary to enhance the attention again to modulate the pictures at later stages, resulting in failing in observing the uncertainty effect in N2.

However, our findings were inconsistent with a previous study by Yang et al. (2012), which showed greater P2 and smaller N2 amplitudes for uncertain compared to certain fearful faces. One possible reason for the discrepancies may be related to stimuli serving as cues. In Yang et al.'s (2012) study, simple neutral symbols (e.g., “+”) and emotional pictures served as uncertain and certain cues, respectively. In the fearful face condition, uncertain and certain cues were different not only in the meanings but also in some other factors, such as the emotional contents and composition of the cues. A recent study by Guan et al. (2015) reported that the P2 to target faces was reduced by emotional compared to neutral primes. Therefore, it is possible that P2 amplitudes for faces were also reduced by the emotional contents of certain cues in Yang et al.'s (2012) study, which resulted in altering the uncertainty P2 effects. In addition, stimuli with complex composition are found to enhance N2 amplitudes (Wiens et al., 2011). As the composition was more complex for certain cues (emotional pictures) compared to uncertain cues (simple symbols) in Yang et al.'s (2012) study, the uncertainty N2 effects may be altered.

In addition, Gole et al. (2012) did not report the uncertainty P2 effect, regardless of the emotion. One possible reason is that the anticipatory intervals were long (6 s) in Gole et al.'s (2012) study. Such long intervals may allow participants to have an appropriate preparation even in the uncertain condition (Lin et al., 2014a), resulting in reducing the uncertainty effect at early sensory stages.

Early LPP (350–450 ms; often overlapping with P3), which is widely distributed over frontal, central, and parietal scalp sites, is supposed to reflect motivated attention (e.g., Nieuwenhuis et al., 2005; Olofsson et al., 2008). High motivated stimuli (i.e., attractive stimuli) were shown to enhance early LPP (Marzi and Viggiano, 2010; Righi et al., 2014). Therefore, our findings indicate that uncertain as compared to certain positive pictures capture more motivated attention. Anselme et al. (2010) proposed that uncertainty enhances the attractiveness of the consequences and motivated attention as a result. Specifically, when people are certain that a positive stimulus is upcoming, they begin to adapt to it, primarily by reaching an understanding of what the stimulus means and why it occurs, and as a result, the stimulus loses some of its force. On the contrary, without knowing the exact emotion and adapting to it in advance, the stimulus produces strong attractive affect, which results in enhancing the motivated attention (Whitchurch et al., 2011).

For negative pictures, we did not find the effect of uncertainty on the early LPP. According to previous studies (e.g., Nieuwenhuis et al., 2005; Olofsson et al., 2008), the findings indicate that motivated attention is similar to uncertain as compared to certain negative pictures. One possible reason is that negative pictures are dangerous and threatening and thus, are perceived as unattractive regardless of the uncertainty.

The late LPP develops around 500 ms after the onset of a stimulus and sometimes lasting for a few seconds. While this component is repeatedly shown to be largest over parietal sites, the anticipation effect seemed to be reflected over anterior sites (Lin et al., 2012; Richards et al., 2013). The late LPP has been found to reflect attentional allocation during the evaluation processes, with larger amplitudes for emotional pictures that are emotional as compared to non-emotional evaluation (Hajcak et al., 2006). Accordingly, our findings may imply that certain compared to uncertain negative pictures are evaluated more negatively and thereby, capture more attentional resources. Previous studies suggested that stimuli are evaluated more negatively when the stimuli are preceded by negative stimuli than when they are preceded by neutral stimuli (de Jong et al., 1995; Tomarken et al., 1995). In the present study, while both certain and uncertain cues were used by neutral symbols; cues definitely indicating the upcoming negative pictures may be perceived as negative in some extent, as these cues have been found to activate the brain regions (e.g., amygdala) associated with negative emotion (e.g., Onoda et al., 2008). In this case, certain cues may enhance the negative evaluation to the negative pictures and the attention as a result.

However, we did not find the effect of uncertainty on the late LPP to positive pictures. Therefore, our findings may indicate that uncertainty does not alter the evaluation and the attention toward positive pictures as a consequence. While according to the above-described studies (de Jong et al., 1995; Tomarken et al., 1995), preceding certain cues may enhance the positive evaluation toward positive pictures; the positive evaluation toward the pictures may also be enhanced by uncertainty (Wilson et al., 2005). In this case, differential positive evaluation toward uncertain as compared to certain positive pictures may be decreased, resulting in showing similar attention toward these two pictures.

While the present study found that emotional valence of the pictures altered the uncertainty ERP effects, it is still unclear whether the uncertainty effect can be modulated by the arousal of the pictures or not. If this is really the case, then is the uncertainty effect modulated by both of the factors? Further studies should be devoted to investigate the issues in more detail.

## Conclusion

The present study showed that negative pictures evoked smaller P2 and late LPP and larger N2 amplitudes in the uncertain as compared to the certain condition. For positive pictures, early LPP was larger in the uncertain as compared to the certain condition. Taken together, our findings indicate that uncertainty about the emotional contents of the pictures modulates the attention to the pictures, and that this modulation is altered by the valence of the pictures.

## Author contributions

HL was involved in study design, execution, data analysis, and manuscript drafting and revises. HJ was involved in study design and manuscript revises. JL, RY, TL, and YW were involved in data analysis and manuscript revises. We have read and approved the manuscript and agree to be accountable for all aspects of the work in ensuring that questions related to the accuracy or integrity of any part of the work are appropriately investigated and resolved.

### Conflict of interest statement

The authors declare that the research was conducted in the absence of any commercial or financial relationships that could be construed as a potential conflict of interest.

## Footnotes

^1^The early CNV, which starts appropriately 1 s after the cue onset and is maximal at frontal-central scalp sites, is often related to the alerting and attention properties of the cue (e.g., Weerts and Lang, 1973; Rohrbaugh et al., 1976; Sakamoto et al., 2009). More importantly, this early CNV component has been found to reflect the processing of uncertainty during the anticipation phase, with reduced amplitudes when the task requirement was uncertain (Linssen et al., 2013). Therefore, it is important to investigate the relationships between this component and emotionally uncertain cues. For more detail, please refer to our previous study (Lin et al., 2014a).

^2^As the alpha ERD/ERS was calculated by individual alpha frequency (IAF), the bandwidth ranges of lower-1, lower-2, and upper alpha were different across participants.
